# Heritability of Addison’s disease and prevalence of associated autoimmunity in a cohort of 112,100 Swedish twins

**DOI:** 10.1007/s12020-017-1441-z

**Published:** 2017-10-16

**Authors:** Jakob Skov, Jonas Höijer, Patrik K. E. Magnusson, Jonas F. Ludvigsson, Olle Kämpe, Sophie Bensing

**Affiliations:** 1 Department of Molecular Medicine and Surgery, Karolinska Institutet, 17176 Stockholm, Sweden; 2 Institute of Environmental Medicine, Karolinska Institutet, Stockholm, Sweden; 3 Department of Medical Epidemiology and Biostatistics, Karolinska Institutet, Stockholm, Sweden; 4 Center for Molecular Medicine, Department of Medicine (Solna), Karolinska Institutet, Stockholm, Sweden

**Keywords:** Addison’s disease, Autoimmunity, Heritability, Polyglandular, Registries, Twins

## Abstract

**Purpose:**

The pathophysiology behind autoimmune Addison’s disease (AAD) is poorly understood, and the relative influence of genetic and environmental factors remains unclear. In this study, we examined the heritability of AAD and explored disease-associated autoimmune comorbidity among Swedish twins.

**Methods:**

A population-based longitudinal cohort of 112,100 Swedish twins was used to calculate the heritability of AAD, and to explore co-occurrence of 10 organ-specific autoimmune disorders in twin pairs with AAD. Diagnoses were collected 1964–2012 through linkage to the Swedish National Patient Register. The Swedish Prescribed Drug Register was used for additional diagnostic precision. When available, biobank serum samples were used to ascertain the AAD diagnosis through identification of 21-hydroxylase autoantibodies.

**Results:**

We identified 29 twins with AAD. Five out of nine (5/9) monozygotic pairs and zero out of fifteen (0/15) dizygotic pairs were concordant for AAD. The probandwise concordance for monozygotic twins was 0.71 (95% CI 0.40–0.90) and the heritability 0.97 (95% CI 0.88–99). Autoimmune disease patterns of monozygotic twin pairs affected by AAD displayed a higher degree of similarity than those of dizygotic twins, with an incidence rate ratio of 15 (95% CI 1.8–116) on the number of shared autoimmune diagnoses within pairs.

**Conclusions:**

The heritability of AAD appears to be very high, emphasizing the need for further research on the genetic etiology of the disease. Monozygotic twin concordance for multiple autoimmune manifestations suggests strong genetic influence on disease specificity in organ-specific autoimmunity.

## Introduction

Autoimmune Addison’s disease (AAD) accounts for close to 90% of all cases of primary adrenal insufficiency in developed countries today [1]. A hallmark of AAD is the presence of 21-hydroxylase (21-OH) autoantibodies. Such antibodies are usually detectable months or years before onset of clinical symptoms and often persist decades after complete adrenocortical destruction [2]. Upon diagnosis, lifelong replacement therapy consisting of glucocorticoids (hydrocortisone or cortisone acetate) and mineralocorticoids (fludrocortisone) is commenced [3].

AAD may appear in isolation, but it is more often part of an autoimmune polyendocrine syndrome (APS) [4]. APS-1 is a rare monogenic disorder caused by mutations in the autoimmune regulator gene (AIRE) [4]. Clinical manifestations typically includes AAD, hypoparathyroidism, and chronic mucocutaneous candidiasis. Most cases of AAD however, present either as part of autoimmune polyendocrine syndrome type 2 (APS-2), or as isolated disease. In APS-2 the patient suffers from two or more organ specific autoimmune manifestations. Autoimmune thyroiditis with hypothyroidism, type-1 diabetes and vitamin B-12 deficiency secondary to autoimmune atrophic gastritis are the most frequent disorders in conjunction with AAD, but many other endocrine and non-endocrine diseases have been reported [1]. APS-2 is sometimes further subdivided according to phenotypic presentation into APS-3 and APS-4 [1].

AAD is most common in the Nordic countries, but it is still a rare disease with published prevalence estimates ranging from 14.4 per 100,000 in Norway to 22.1 per 100,000 in Iceland [5]. The prevalence in Sweden appears to be similar to that in Norway with one study identifying 1305 patients in a population of 9 million [6].

AAD without mutations in the AIRE gene does not follow Mendelian patterns of inheritance. It is multifactorial in origin and under influence of several genetic variants, only some of which have so far been mapped [7–9]. No environmental factors predisposing for AAD have been identified and the relative importance of environmental vs. genetic factors is unknown.

Familial aggregation of AAD is evident from previous studies. In a cohort of 660 Norwegian patients with AAD, 10% had another family member (first to third-degree relative) with AAD [10] and in a Swedish study, 6.4% of the patients with AAD without APS-1 reported having a family member (sibling, parent, child, or relative) with AAD [11]. Published data on twins is limited to anecdotal case reports on identical twin concordance [12–15], and no formal studies evaluating the genetic influence on AAD have been performed to date. In this study, we aimed to investigate concordance and heritability for AAD in a large population-based twin cohort with high disease prevalence.

## Subjects and methods

### Registries

The Swedish Twin Registry (STR) is one of the world’s largest twin resources. It was started in 1961 and to date contains information on more than 194,000 individual twins born in Sweden between 1886 and 2007, with nationwide coverage (≈ 95%) since 1942. Zygosity has been determined for more than 75,000 twin pairs using a validated intra-pair similarity algorithm, DNA or opposite sex [16]. Biobank material including serum is available for 15,000 twins. The Swedish National Patient Register (NPR) contains inpatient information dating back to 1964, with nationwide coverage since 1987. It includes hospital admission dates, discharge dates, discharge codes and procedural coding for surgery classified according to the International Statistical Classification of Diseases (ICD). As of 2001 data on outpatient care are also included. The Prescribed Drug Register (PDR) collects data on all prescription drugs dispensed in Sweden from July 2005 onwards.

### Serologic testing

A full-length cDNA clone was used to manufacture 35S-radiolabeled 21-hydroxylase antigen with the TNT system (Promega). This was subsequently immunoprecipitated with patient sera. Blood donors served as negative controls and sera from APS-1 patients with known strong reactivity to 21-hydroxylase were used as positive controls. Radioactivity was measured using a liquid scintillation counter (Wallac 1450 MicroBeta, Perkin-Elmer). The cutoff value was set to obtain maximal accuracy, based on the results from a recent inter-laboratory study[17].

### Study population

**Fig. 1:**
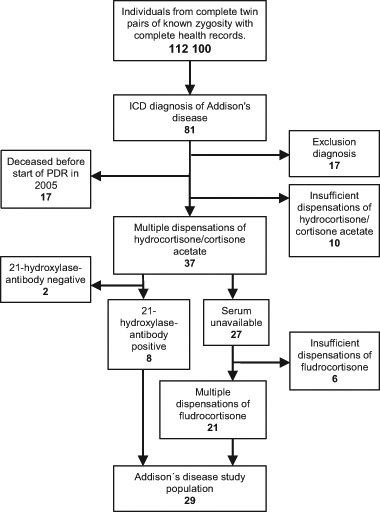
Case ascertainment for autoimmune Addison’s disease. ICD—International Statistical Classification of Diseases. PDR—Prescribed Drug Register

By combining information from the STR and the NPR, we could retrieve health records on 112,100 twins of known zygosity. Twins diagnosed with Addison’s disease (ICD-7: 274.40, ICD-8: 255.10, ICD-9: 255E, ICD-10: E27.1, E27.2) from 1964 to 2012 were identified in the NPR. To rule out adrenal failure from causes other than AAD, as well as monogenic AAD, patients with diagnoses of (1) any pituitary disease or Cushing’s syndrome, (2) neoplasm of the adrenal glands, (3) adrenogenital disorders, (4) adrenoleukodystrophy, (5) drug-induced adrenocortical insufficiency, (6) adrenal hemorrhage or infarction, (7) unspecified adrenocortical insufficiency, (8) post procedural adrenocortical hypofunction, (9) tuberculosis, (10) HIV disease, (11) Waterhouse-Friderichsen syndrome or, (12) primary hypoparathyroidism were excluded (see Online Resource 1 for ICD-codes) To further improve accuracy, patients lacking multiple (≥2) dispensations of hydrocortisone or cortisone acetate (ATC H02AB09/H02AB10) in the PDR were excluded. Consequently, all patients deceased before the start of the PDR in July 2005 were excluded. For the remaining cohort, biobank serum samples were used when available if samples had been drawn after a diagnosis of Addison’s disease, for analysis of 21-OH autoantibodies. Individuals with positive titers were classified as having AAD and were subsequently included in the final patient cohort; twins with negative titers were considered as not having AAD and were therefore excluded from further analysis. For patients without biobank serum samples, multiple (≥2) dispensations of fludrocortisone (ATC H02AA02) were required for inclusion in the final cohort. The ascertainment process is outlined in Fig. 1.

Diagnoses for the ten most common autoimmune manifestations found in association with AAD in APS-2 [1, 4, 10] (Table 1) were identified in the NPR for all twin pairs with AAD. Autoimmune atrophic gastritis and pernicious anemia are ICD-coded separately but were considered as the same autoimmune disease. The PDR was used for additional diagnostic precision and coverage regarding APS-2 manifestations among patients alive after December 2005. Multiple dispensations of short acting insulin/insulin analogs (ATC A10AB) were required for a diagnosis of type-1 diabetes, multiple dispensations of levothyroxine (ATC H03AA) for a diagnosis of Hashimoto’s thyroiditis and multiple prescriptions of vitamin B12 (ATC B03BA) for a diagnosis of pernicious anemia. Both Hashimoto’s thyroiditis and atrophic gastritis are frequently not ICD-coded in conjunction with AAD. Thus, patients with multiple prescriptions of levothyroxine without a diagnosis indicating other thyroid disorders were classified as having Hashimoto’s thyroiditis, and patients with multiple prescriptions of vitamin B12 without a diagnosis of bariatric surgery, gastric resection or malabsorption were classified as having autoimmune atrophic gastritis (see Online Resource 1 for ICD-codes and procedure-codes).

**Table 1:** Clinical characteristics of the twin cohort and Scandinavian population based cohorts

Clinical characteristics	Twin cohort	Norwegian cohort [12]	Swedish cohort [15]
	(*n* = 29)	(*n* = 426)	(*n* = 660)
Mean age at onset	38	39	34
Female (%)	15 (52%)	64%	59%
Autoimmune comorbidity (%)	18 (62%)	66%	62%
Autoimmune thyroid disease (%)	14 (48%)	47%	47%
Hashimoto thyroiditis (%)	13 (45%)	41%	40%
Grave’s disease (%)	1 (3%)	6%	7%
Pernicous anemia/atrophic gastritis (%)	4 (14%)	12%	11%
Type-1 diabetes (%)	3 (10%)	10%	11%
Vitiligo (%)	2 (7%)	11%	6%
Celiac disease (%)	1 (3%)	3%	2%
Ovarian or testicular failure	0	4%	4%
Alopecia	0	4%	^a^

^a^ Data not available

### Statistical methods

Different heritability models were estimated, with the aim of investigating the magnitude of the genetic and environmental components. The genetic factors are additive (A) and dominant (D), whereas the environmental factors are shared (C) and unique (E). Heritability was defined as the variance of the genetic factor(s) divided by the total variance in the liability threshold models. Using structural equation modeling, the three liability threshold models AE, ACE, and ADE were estimated [18]. For each of the models, Akaike information criteria (AIC) were calculated, and the model with the lowest AIC was chosen as the preferred model.

Probandwise concordance was defined as the of probability of AAD, given AAD in the co-twin. Concordance rate was calculated using logistic regression with cluster robust standard errors to account for dependencies within each pair. Poisson regression with cluster robust standard errors was used when comparing autoimmune comorbidity between males/females and between zygosities. In these analyzes, age at end of study or death was used as exposure time.

When comparing continuous variables between groups, linear regression with cluster robust standard errors was used for mean values, and median regression [19] with cluster robust standard errors was used for median values, with both methods treating the twin pairs as clusters. APS-2 concordance was quantified by counting the number of concordant APS-2 diagnoses within each twin pair. To assess the difference in APS-2 concordance between MZ and DZ twins, we used Poisson regression, treating each pair as a unit. Since the twins and the twin pairs had been in the study for different periods of time, we used each pair’s mean time in the study as exposure time for calculating rates.

Two-sided tests were used with 5% significance level. We used the statistical programs Stata (StataCorp), v.13, and R (R Foundation for Statistical Computing), v.3.2.2.

## Results

The Swedish twin cohort included a total of 112,100 individuals, 36,650 of whom were part of monozygotic twin pairs, 47,500 were part of same sex dizygotic twin pairs and 27,950 were part of opposite sex dizygotic twin pairs. Among them we identified 29 individuals (15 women and 14 men) with AAD (prevalence 25.9 per 100,000). The median age at diagnosis was 40 years (range 27–72 years) among women and 29 years (range 8–67 years) among men. Most patients (10 women, 8 men) had one or more autoimmune comorbidity, with Hashimoto’s thyroiditis (*n* = 13), pernicious anemia (*n* = 4), and type-1 diabetes (*n* = 3) being the most common. Co-morbidities in two out of the 13 patients with hypothyroidism and 3 out of the 4 patients with pernicious anemia were identified by prescriptions using the PDR. There were no recorded cases of primary ovarian failure, testicular hypofunction or alopecia areata. There was no significant difference in prevalence of autoimmune comorbidity between women and men (mean 0.9 vs. 0.7 manifestations, *p* = 0.83). Overall prevalence of APS-2 manifestations was similar to that previously reported in larger cohorts [10, 11] (Table 1).

A total of 9 MZ and 15 DZ twin pairs were affected by AAD. Among MZ twins five pairs (four female, one male) were concordant, and four pairs (two female, two male) were discordant for AAD. The mean discordance time in concordant twin-pairs was 14 years (range 4–31). In discordant MZ pairs, the mean time of follow-up from diagnosis of AAD was 30.7 years (range 17–42). All 15 DZ pairs (six same sex male pairs, three same sex female pairs, six opposite sex pairs) were discordant for AAD. The mean time of follow up from diagnosis of AAD was 17.6 years (range 0.3–42) in DZ twins. MZ and DZ twins with AAD did not differ significantly in clinical characteristics apart from sex (See Online Resource 1). Probandwise concordance among MZ twins was estimated at 0.71 (95% CI 0.40–0.90). Probandwize concordance appeared to be higher in MZ females (0.80 95% CI 0.42–0.96) than in MZ males (0.5, 95% CI 0.08–0.92), but the difference was not statistically significant (*p* = 0.32). Using AIC calculations, the AE-model provided the best overall fit for the data, with 97% of disease liability attributable to additive genetic effects, and 3% to unique environment not shared by co-twins. This translates to a heritability estimate of 0.97 (95% CI 0.88–0.99).

**Fig. 2:**
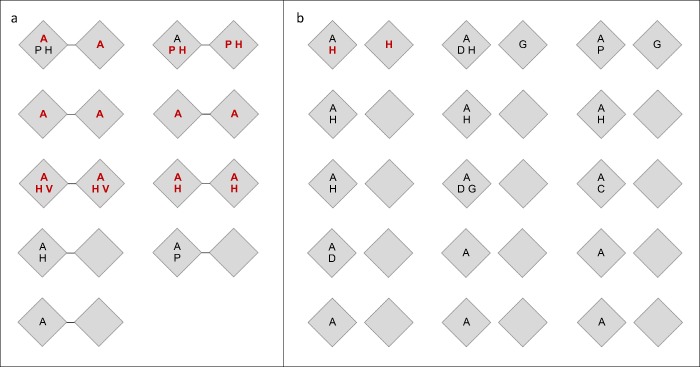
Patterns of autoimmunity in twin pairs with autoimmune Addison’s disease. Organ-specific autoimmune prevalence in monozygotic **a** and dizygotic **b** twin pairs with autoimmune Addison’s disease. (A) Addison’s disease, (H) hypothyroidism, (G) Grave’s disease, (D) Type-1 diabetes, (C) celiac disease, (P) pernicous anemia, (V) vitiligo. Concordant diagnoses in bold red letters. Disease patterns are unique twin pairs, to conceal identity sex is not displayed

Taking all APS-2 manifestations including AAD into account, 20 of 28 cases of autoimmune disease recorded in the nine MZ pairs displayed concordance, i.e., the same disease was present in both twins (Fig. 2). DZ pairs displayed 27 cases of autoimmune disease in the 15 twins affected by AAD, and one APS-2 manifestation each in three co-twins without AAD, with disease concordance for two of 30 manifestations. The difference in APS-2 concordance between MZ and DZ twins was statistically significant with an incidence rate ratio of 15 (95% CI 1.8–116) using Poisson regression on the number of concordant diagnoses within the twin pairs.

## Discussion

We have used the population-based STR to identify most, if not all, Swedish twins with AAD. With an estimated heritability of 0.97 (95% CI 0.88–0.99) our data indicate strong genetic influence on the occurrence of disease. Previous reports have demonstrated substantial heritability for organ-specific autoimmune disorders such as type-1 diabetes (0.72–0.88) [20, 21] and autoimmune thyroid disease (0.73–0.79) [22, 23]. Heritability for celiac disease is estimated at 0.58–0.87 [24, 25], with probandwise MZ concordance rates of 0.49–0.83—among the highest reported for any autoimmune disorder under the influence of complex traits [26]. We observe similar MZ concordance rates, but for a disease 20 times more infrequent. This indicates major familial influence, and without DZ twin concordance most of this risk is likely to be attributable to genetic components. HLA DR3-DQ2 and DR4 are major risk factors for AAD [10]. Besides the strong HLA association, several loci involved in immune system regulation have been linked to AAD [8, 10, 27]. Most, but not all [9], have been identified through candidate-gene studies on risk loci implicated in other organ specific autoimmune disorders common in APS-2. The genetic risk factors so far identified cannot account for more than a minor fraction of the total heritability observed, emphasizing the need for large scale genetic analysis on AAD to identify additional risk loci. Dominance or epistatic effects [28], i.e., that the combination of two risk alleles is higher than the additive effect of each, may also contribute to the high heritability observed in AAD. An obvious example may be that MHC risk haplotypes only exert their effect in conjunction with other risk alleles.

Autoimmune disease tends to cluster in both individuals and families, with multigenerational pedigrees often displaying a variety of manifestations [29, 30]. This likely reflects the fact that most of the genetic liability to autoimmunity can be attributed to MHC and polymorphisms involved in immune regulation, with pleiotropy across diseases, and not to the target organs themselves [31, 32]. To what extent unidentified genetic factors, environmental triggers or stochastic events determine which disease will develop in patients who are prone to autoimmunity is still unknown. What sets AAD apart from most other disorders is the high frequency of autoimmune comorbidity. In Scandinavian population-based cohorts 60–66% of patients have other autoimmune manifestations [6, 10, 11], with a plethora of different phenotypes. Based on these observations we expected diverse patterns of comorbidity both within and between twin pairs. Observations in DZ twins were limited by low autoimmune burden in the non-Addison twin, but did not conflict with this assumption. Surprisingly, MZ twins displayed similar disease patterns within pairs, suggesting strong genetic influence not only on AAD but also on the disease specificity of associated autoimmune disorders, with little influence from stochastic processes on what disease(s) patients eventually develop. A possible explanation for our observation is that genetic variants interact to produce discrete risk profiles that predispose to certain diseases, but not to others. These profiles are identical in MZ twins but vary between DZ twins and other first degree relatives. Whether this observation is correct only in conjunction with AAD or holds true in other autoimmune polyendocrine syndromes remains to be examined, but phenotypical similarities in MZ twins have been observed in other autoimmune conditions. In Crohn’s disease clinical characteristics are often comparable in MZ twins [33]. In type-1 diabetes discordance time in MZ twins is generally much shorter than in DZ twins, indicating genetic influence on disease progression [21, 34]. AAD appears to differ in this aspect, with discordance times ranging from 4–31 years in our study.

Swedish health registries offer excellent conditions under which to study rare diseases, but misclassifications do occur, and secondary adrenal insufficiency is sometimes miscoded as AAD. Accordingly, we used rigorous exclusion criteria, but still, as we in the twin cohort were lacking serum samples for 21-OH autoantibody screening from some twin pairs, we cannot completely rule out the presence of non-autoimmune Addison’s disease. We may also have lost true cases of AAD, as twins lacking fludrocortisone were excluded despite the fact that approximately 10% of patients in the Swedish Addison registry do not use mineralocorticoids [11]. If treating physicians were more vigilant in searching for, and coding for, diseases present in MZ counterparts, that may have inflated or distorted concordance scores. Incomplete coding of “lesser” comorbidities may also have affected our results. We did circumvent this problem to some extent by using the PDR, but for diagnoses without specific treatment, such as alopecia and vitiligo, that was not possible.

A common misconception of heritability is that estimates approaching 1 as reported in our study equates to near Mendelian patterns of inheritance. That is not the case, heritability explains the proportion of variation in a given population that can be attributed to genetic variations in that same population. Thus, a high heritability estimate is not the opposite of phenotypic plasticity, nor does it exclude environmental influence on the occurrence of disease, but it does point to significant genetic contributions. Furthermore, the heritability models used here do not account for underlying gene-environment interactions or epistatic effects [35], both of which could potentially inflate the additive genetic effect.

Finally, small patient cohorts will always limit twin studies on rare diseases, and our study is no exception. Improved estimates would however, require a larger patient cohort, and that is unlikely to emerge, given that we have used a large twin resource to examine a high prevalence population.

In summary, we demonstrate strong heritability for AAD, underscoring that many risk alleles remain to be identified. Monozygotic twin concordance for multiple autoimmune manifestations suggests strong genetic influence on disease specificity, despite the pleiotropic nature of many risk alleles associated with autoimmune disease.

## Electronic supplementary material

Online Resource 1
