# Supplementary material for: Heritability of Addison’s disease and prevalence of associated autoimmunity in a cohort of 112,100 Swedish twins
Source: Endocrine. 2017 Oct 16;58(3):521–7. doi: 10.1007/s12020-017-1441-z (PMC5693969; doi:10.1007/s12020-017-1441-z)
Supplement: Supplementary file 1 — Online Resource 1 [file 12020_2017_1441_MOESM1_ESM.docx]

**Online Resource 1:**

**Inclusion and exclusion criteria for Addison’s disease**

Inclusion criteria:

ICD-10 E27.1, E27.2

ICD-9 2554

ICD-8 255,10

ICD-7 274.40

AND

Multiple dispensations of hydrocortisone/cortisone acetate ATC H02AB09/H02AB10 in SPDR in combination with either 21-OH antibody positivity or with multiple dispensations of fludrocortisone ATC H02AA02.

Exclusion criteria:

|  | ICD10 | ICD9 | ICD8 | ICD7 |
| --- | --- | --- | --- | --- |
| Tuberculosis | A15-A19, P37.0, J65, B90 | 010-018, 137 | 010-019, Y34.09, Y34.19, Y34.29 | 001.99-019.20, Y03.00, Y03.10, Y03.20, Y53.00 |
| Waterhouse-Friedrichsens syndrome | A39.1 | 0363 | 036.11 | 057.11 |
| HIV | B20-B24 | 0798, 2799 |  |  |
| Malignant neoplasm of adrenal gland | C74, C79.7 | 1940, 1987 | 194.01 | 195.01 |
| Benign neoplasm of adrenal gland | D35.0 | 2270 | 226.00-09 | 224.10-224.19 |
| Neoplasm of uncertain or unknown behaviour of adrenal gland | D44.1 | 2372 | 239.10 | 195.00, 239.31 |
| Adrenogenital disorders | E25 | 2552 | 255.01, 255.02 |  |
| Drug-induced adrenocortical insufficiency | E27.3 |  |  |  |
| Other and unspecified adrenocortical insufficiency | E27.4 | 2555 |  |  |
| Disorders of adrenal glands in diseases classified elsewhere  (TB A18.7, meningococcal A39.1) | E35.1 | 2555 |  |  |
| Adrenoleukodystrophy | E71.3 | 3300 | 333.10 |  |
| Postprocedural adrenocortical(-medullary) hypofunction | E89.6 | 2555 |  |  |
| Neonatal adrenal haemorrhage | P54.4 | 7725 |  |  |
| Congenital malformations of adrenal gland | Q89.1 | 7591 | 758.10 |  |
| Hyperfunction of pituitary gland | E220, E222, E228, E229 | 253* | 253* | 272* |
| Hypofunction and other disorders of pituitary gland | E23* | 253* | 253* | 272* |
| Cushing syndrome | E24* | 2550 | 258.00 | 277.10 |
| Malignant neoplasm of pituitary gland | C75.1 | 1943 | 194.31 | 195.31 |
| Benign neoplasm of other pituitary gland | D35.2 | 2273 | 226.20 | 195.30, 224.40 |
| Neoplasm of uncertain or unknown behaviour of pituitary gland | D44.3 | 2370 | 239.90 | 239.34 |
| Postprocedural hypopituitarism | E89.3 | 2537 |  |  |

* all codes

**Inclusion and exclusion criteria for 10 most common diagnoses found in association with autoimmune Addison’s disease**

**Hashimoto’s thyroiditis**

Inclusion criteria:

ICD-10 E035 E039 E063 E065

ICD-9 2449 2452 2458

ICD-8 244.09 245.03

ICD-7 253.00 253.19 253.29 254.00

Exclusion criteria:

ICD-10 E030 E031 E032 E033 E064 E890 E00 E01 E02 E060 E061

ICD-9 2461 243 2440 2441 2442 2443 2448

ICD-8 243.99 245.00 245.01 245.02 245.04 244.00

ICD-7 250.00 253.10 253.20 254.10 254.20

(procedural codes)

BAA60 BAA40 BAA50 BAA20

810 811 814 820 812 813 822 824 830

No dispensations of H03AA in SPDR despite being alive after July 1^st^ 2006.

Alternative for diagnosis:

Part of Addison’s disease twin set with multiple dispensations of levothyroxine (H03AA) in SPDR without:

Diagnosis of Grave’s disease

Diagnosis of thyrotoxicosis (E059 (ICD10), 242 (ICD9), 242.20 (ICD8))

Exclusion diagnosis for Grave’s disease

Exclusion diagnosis for Hashimoto’s thyroiditis

**Pernicious anemia**

Inclusion criteria:

ICD-10 D510

ATC-code B03BA

ICD-9 2810

ICD-8 281.00 281.09

ICD-7 290.00 290.10

Exclusion criteria:

procedural codes

JDC, JDF, 4411-4435 4439

**Atrophic gastritis**

Inclusion criteria:

ICD-10 K293 K294 K295

ICD-9 5351

ICD-8 535.03

ICD-7 543.01

Exclusion criteria:

none

**Type-1 diabetes**

Inclusion criteria:

ICD 10 E10

ATC code for insulin A10A

ICD 8-9 250 and age ≤30

ICD 7 260 and age ≤30

Exclusion criteria

ICD-10 More E11 than E10 diagnoses

**Grave’s disease**

Inclusion criteria:

ICD-10 E050 E055

ICD-9 2420

ICD-8 242.00 242.09

ICD-7 252.00 252.01 252.02

Exclusion criteria:

ICD 10 E051 E052 E053 E054 E058 E060 E061

ICD-9 2421 2422 2424 2428 2450 2451

ICD-8 242.10 245.00 245.01 245.02

ICD-7 252.10 254.10 254.20

**Celiac disease**

Inclusion criteria:

ICD-10 K900

ICD-9 5790

ICD-8 269.00 269.98

ICD-7 286.00

Exclusion criteria:

Lacking ICD-9 code 5790 and ICD-10 code K90.0 if alive 1997 (=10 years with specific code for celiac disease in ICD9/10)

**Primary ovarian failure**

Inclusion criteria:

ICD-10 E283 and age <40

ICD-9 2564 and age <40

ICD-8 256.10 or 627.02 and age <40

ICD-7 275.00 and age <40

Exclusion criteria:

ICD-10 E282 Q96

ICD-9 2564 2563 7586

ICD-8 256.90 759.50

ICD-7 275.20 275.10

**Testicular hypofunction**

Inclusion criteria:

ICD-10 E291

ICD-9 2572

ICD-8 257.10

ICD-7 276.00

Exclusion criteria:

No dispensations of G03B in PDR despite being alive after July 1^st^ 2006.

ICD-10 Q98

ICD-9 7587 2571

ICD-8 759.51

ICD-7 276.10

**Alopecia areata**

Inclusion criteria:

ICD-10 L63

ICD-9 7040

ICD-8 704,00

ICD-7 713.03 713.04

Exclusion criteria:

ICD-10 L64 L650 L651 L652 L658 L66

ICD-9 none

ICD-8 none

ICD-7 713.00 713.01 713.02

**Vitiligo**

Inclusion criteria:

ICD-10 L80

ICD-9 7090

ICD-8 709.05

ICD-7 716.11

Exclusion criteria:

none

**Supplemental table 1**: Clinical characteristics of monozygotic and dizygotic twins with Addison’s disease.

|  | **Monozygotic twins** | **Dizygotic twins** | **P-value** |
| --- | --- | --- | --- |
| Number of patients | 14 | 15 |  |
| Female (%) | 10 (71%) | 5 (33%) | 0.05 |
| Median age at diagnosis (range) | 36 (19-67) | 31 (8-72) | 0.62 |
| Median time of follow-up from diagnosis of AAD (range) | 19 (3-43) | 14 (0.3-42) | 0.41 |
| Median age at end of follow-up or death (range) | 70 (36-82) | 57 (20-76) | 0.14 |
| Mean number of autoimmune comorbidities (SD) | 0.9 (0.9) | 0.8 (0.7) | 0.93 |
